# Recent Advances in Electrochemical Biosensors for the Detection of *Salmonellosis*: Current Prospective and Challenges

**DOI:** 10.3390/bios12060365

**Published:** 2022-05-26

**Authors:** Subhasis Mahari, Sonu Gandhi

**Affiliations:** 1 DBT-National Institute of Animal Biotechnology (NIAB), Hyderabad 500032, India; maharisubhasis@gmail.com; 2 DBT-Regional Centre for Biotechnology (RCB), Faridabad 121001, India

**Keywords:** electrochemical biosensors, point-of-care, *Salmonella*, transducer, detection

## Abstract

Salmonellosis is a major cause of foodborne infections, caused by *Salmonella*, posing a major health risk. It possesses the ability to infiltrate the food supply chain at any point throughout the manufacturing, distribution, processing or quality control process. *Salmonella* infection has increased severely and requires effective and efficient methods for early monitoring and detection. Traditional methods, such as real-time polymerase chain reaction and culture plate, consume a lot of time and are labor-intensive. Therefore, new quick detection methods for on-field applications are urgently needed. Biosensors provide consumer-friendly approaches for quick on-field diagnoses. In the last few years, there has been a surge in research into the creation of reliable and advanced electrochemical sensors for the detection of *Salmonella* strains in food samples. Electrochemical sensors provide extensive accuracy and reproducible results. Herein, we present a comprehensive overview of electrochemical sensors for the detection of *Salmonella* by focusing on various mechanisms of electrochemical transducer. Further, we explain new-generation biosensors (microfluidics, CRISPR- and IOT-based) for point-of care applications. This review also highlights the limitations of developing biosensors in *Salmonella* detection and future possibilities.

## 1. Introduction

*Salmonella* is one of the most prevalent bacteria causing foodborne illnesses and death [1]. *Salmonella* is a Gram-negative, rod-shaped bacterium which belongs to the Enterobacteriaceae family [2,3]. Common standard methods for the identification of *Salmonella* bacteria are based on culture techniques, enzyme-linked immunosorbent assay (ELISA) and nucleic acid-based approaches [4]. Nucleic acid approaches include polymeric chain reaction (PCR), loop-mediated isothermal amplification (LAMP), nucleic acid sequence-based amplification (NASBA), micro arrays, recombinase polymerase amplification (RPA), and whole-genome sequencing (WGS). Some of these techniques require highly skilled individuals and sophisticated apparatus, and others consume a lot of time. As a result, the development of specific, sensitive and reliable technologies for quick detection is always needed for *Salmonella* diagnosis. Biosensors provide a number of advantages over laboratory-based assays, including increased sensitivity accuracy and specificity as well as the low cost, rapid response, in situ applications and potential for portability [5,6,7,8,9]. As a result, they have been identified as impressive alternatives for detecting *Salmonella* in food. There has been a surge in research investigations in this sector in recent years, with several publications. Numerous studies on this topic have also been published, with specific use of nanomaterials, electrochemical signal interpretation, and aptamer identification [10,11,12,13].

Electrochemical sensors detect the analyte of interest based on potentiometry, conductometry and impedimetric. Electrochemical sensing has been prevalent because of its rapid, specific detection response, sensitivity, ability to be miniaturized and integrated into point-of-care testing [14]. Furthermore, the utilization of different nanomaterials such as carbon nanotubes (CNTs), metallic nanoparticles, silica nanoparticles, metal oxide nanoparticles and organic nanoparticles enhances the detection limit in comparison with sensors with only molecular probes, antibodies or peptides [15]. Therefore, the selection of a bio-recognition element in combination with a nanomaterial is highly essential for electrochemical sensor development that would help with the sensitive and rapid detection of analytes [16].

**Figure 1:**
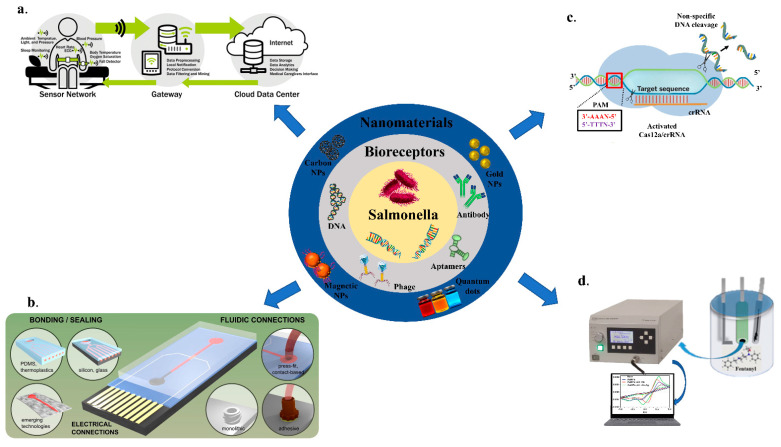
An overview of biosensors for the detection of Salmonella; (**a**) IOT-based; Adapted with permission from Ref. [17]. Copyright 2018, Elsevier. (**b**) Microfluidics based; Adapted with permission from Ref. [18]. Copyright 2015, Elsevier. (**c**) CRISPR/Cas-based; (**d**) Potentiometry-based biosensing.

This review focuses on different electrochemical sensors where we have discussed different bioreceptors and the use of nanomaterials for the detection of *Salmonella*. The review also provides the details of recently developed biosensors for sensing of *Salmonella* in food samples and the measures taken to miniaturize and integrate the sensors into point-of-care applications, including IOT-based (Temiz et al., 2015) [17], microfluidics (Rahmani et al., 2018) [18], CRISPR-based and potentiometric sensors (Figure 1).

## 2. Materials and Methods

PubMed and Web of Science databases were used to search the articles. Electrochemical biosensors, *Salmonella*, point-of care applications, new-generation biosensors, transducers, probes used in sensing *Salmonella*, were the key words used to search while writing the review. Each article was screened thoroughly based on their electrochemical sensing methods in detecting the *Salmonella* pathogen and the probe used in detection. The screening was also carried out to select various nanomaterials used to increase the sensitivity of the biosensors. The cited references were selected on the basis of recent publications in electrochemical sensors and new-generation biosensors to detect *Salmonella*. 

## 3. Emergence of Probe Based Sensing Approaches for *Salmonella*

In the last few years, detection of *Salmonella* has gained popularity due to its increased rate of infection. Specific probes are required for designing a biosensor to detect *Salmonella* since they help in precise, sensitive, and effective detection [19]. Biomolecules or a combination of probes with capabilities to recognize targets can theoretically be utilized as receptors in a biosensor. Aptamers, antibodies, anti-microbial peptides (AMPs) and bacteriophages are essential probes used to detect *Salmonella* [20].

### 3.1. Single-Stranded DNA/RNA-Based Probes for Sensing of Salmonella

Research on single-stranded RNA or DNA (aptamers) generated using in vitro selection methodology called Systematic Evolution of Ligands by Exponential Enrichment (SELEX) was first published in the early 1990s by Gold and Szostak [21]. The synthesis of aptamers for a larger variety of analytes using SELEX is possible for a big range of aptamer-based applications. Furthermore, aptamers are stable, affordable, easy to produce with chemical modifications, and have a low immunogenicity.

**Figure 2:**
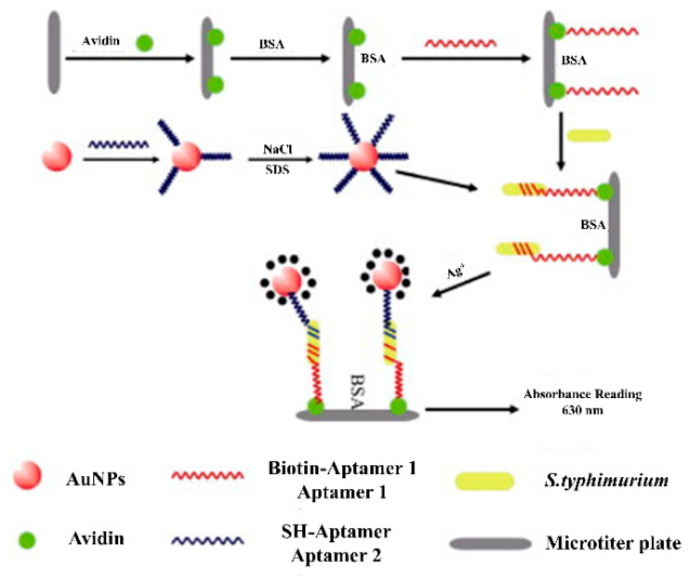
Detection of *Salmonella typhimurium* via attachment of avidin and AuNPs for recognition of biotinylated aptamer 1 and SH-aptamer 2, respectively. Adapted with permission from Ref. [22]. Copyright 2014, Elsevier.

The identification of aptamers, silver signal amplification, nanogold tagging, and a quick, specific, and visible detection approach for *Salmonella typhimurium* was developed in a detection range of 10–10^6^ CFU/mL and LOD of 7 CFU/mL for *Salmonella typhimurium* with high specificity and affinity (Figure 2) [22]. They have evolved into a potent target recognition tool with unrivalled intrinsic benefits such as chemical and physical stability, simplicity of synthetization and modification, non-toxicity, structural memory, and enhanced half-life [23]. When designing several biosensing apparatuses for the detection of *Salmonella*, aptamers have been employed as an alternative to traditional antibodies. Crucially, they have a hybridization ability, with their complementary DNA (cDNA), and they undergo a considerable change in conformation due to the presence of targets, leading to better confirmational changes in the construction of novel biosensors [24,25,26,27]. In a study by Zhang et al., *Salmonella typhimurium* could be detected using a label-free fluorescent silver nanocluster (AgNCs). Herein, a developed sensor system used triple-trigger sequences by regenerating the amplified strand, self-protective hairpin-template-generated scaffolds of AgNCs [28]. In another study by Zhang et al., *Salmonella enteritidis* (*S. enteritidis*) detection was possible using a capture probecomplex. The capture probe complex used the *S. enteritidis*-aptamer and a fluorescent hybridized signal to detect the target. In the presence of the target *S. enteritidis*, single-stranded target sequences were liberated and initiated the replication–cleavage reaction, producing numerous G-quadruplex structures with a linker on the 3’-end confirmed the presence of target [29]. Aptasensors, or ssDNA-based sensors for the diagnosis of *Salmonella* strains, are being used due to their remarkable properties and enormous promise.

### 3.2. Immunoglobulins Based Sensing of Salmonella

Immunoglobulins are generated by the immune system of the body and have enhanced specificity and affinity for its analyte [30]. IgG immunoglobulin comprises two light chains and two heavy chains that are arranged in a Y-shaped configuration. It also has two regions for the activation of immune response, antigen recognition, and fragment antigen-binding region (Fab), and the fragment crystallizable (Fc) region [31]. Antibodies are commonly utilized tool for the detection of *Salmonella* and are considered “gold standard” due to their strong affinity for targets. In research by Raquel et al., electrochemical quantification of the food-borne pathogen *Salmonella enterica serovar typhimurium* using a highly sensitive and label-free laser-induced graphene (LIG) electrode functionalized with antibodies was performed where live Salmonella was detected in chicken soup using LIG biosensors across a wide linear range (25 to 105 CFU/mL) and with a low detection limit of 13 ± 7 CFU/mL [32]. “Nanobody”, a tiny (15 kDa) protein, was designed to overcome these limitations, and being researched for the recognition of Salmonella strains. It is made up of a single heavy-chain variable domain and can be bulk-manufactured using common microbial expression techniques [33]. In a study by Kui et al., specific nanobodies were selected against *Salmonella enteritidis* to develop an improved nanobody–horseradish peroxidase (HRP)-based sandwich ELISA to detect *Salmonella enteritidis* in the real sample [34]. This method does not require a secondary antibody labelled with HRP, thus reducing the time of experimentation and increasing the specificity. Further, the electrochemical detection of *Salmonella* using an Fe3O4@graphene-modified electrode by deposition of gold nanoparticles (AuNPs) was performed by Kaiwan et al. Salmonella typhimurium was detected in a linear range of 2.4 × 10^2^ to 2.4 × 10^7^ CFU/mL, and the limit of detection of the immunosensor was 2.4 × 10^2^ cfu/mL in milk samples [35]. However, isolation and characterization of antibodies from animals are costly, time-consuming, and labor-intensive [36].

### 3.3. Phage-Based Sensing of Salmonella

Phages or bacteriophages are viruses infecting bacteria and replicate using the “machinery” of the host bacterial cells. They have various advantages as novel bioreceptors, resistance to harsh environmental conditions, including excellent selectivity for host bacteria, and the ability to create vast numbers of offspring phages [37]. They have an ability to reproduce inside a living host. Phage-based biosensors can discriminate between dead and living bacteria, which sets them apart from other bioreceptor-based *Salmonella* detection approaches [38,39]. Fernandes et al. used pathogenic phage (PVP-SE1), a broad-spectrum phage as a bio-receptor to discriminate viable and viable-nonculturable (VBNC) *Salmonella* bacterial cells from dead cells, using a broad-spectrum virulent phage (PVP-SE1) as a bioreceptor [40]. Biosensing *Salmonella* has also been described using a variety of bacteriophages, including M13, PRD1, E2, and P22. The detection of *Salmonella* using bacteriophage-based systems is also being commercialized. When the phages were immobilized on the magnetoelastic sensor surface, the response become altered upon exposing it to the target pathogen and measured by a resonance frequency shift. The amount of target antigen attached to the sensor was proportional to the variation in frequency. The sensors tested in water samples with a limit of detection up to 5 × 10^3^ CFU/mL and 159 Hz/decade sensitivity [41,42]. Sample 6 DETECT System (Sample6 Technologies, Inc., Boston, MA, USA) is one of such example, which works by using modified bacteriophages to interface with target bacteria, such as *Salmonella*, and drive them to express luminous enzymes. Anti-salmonella antibodies were attached to horseradish peroxidase (HRP) as an optical reporter and preconcentrated bacteria was obtained by phage-modified magnetic particles. This method achieved exceptional selectivity and sensitivity in milk samples without pre-enrichment, with a detection limit of 19 CFU/mL in 2.5 h [43]. This technology allows for highly sensitive identification of foodborne pathogens, showing results equivalent to immunoassays and polymerase chain reaction (PCR). When bacteriophages are dry, they lose their ability to capture the target, which is a significant disadvantage for them as bioreceptors. Furthermore, host bacterial cells lysis during diagnosis results in a reduction in the recorded signal [44]. In a study by Widjojoatmodjo et al., a magneto immuno PCR assay was developed for identification of *Salmonella* serovars using magnetic particles coated with monoclonal antibody (mAbs) to extract *Salmonella* bacteria. Trapped bacteria were lysed, and the supernatant, which contained bacterial DNA, was subjected to the polymerase chain reaction (PCR) using primers from the *Salmonella typhimurium* to amplify the specific region [45]. In another study by Widjojoatmodjo et al., magnetic immuno PCR assay (MIPA) was developed combining immunomagnetic separation by using specific mAbs and PCR for the direct detection of *Salmonellae* in feces from humans. Immunomagnetically extracted stool samples needed to be diluted only 10-fold when 1 microgram of T4 gene 32 protein was added to the PCR. The MIPA sensitivity obtained was 10^5^ CFU/mL [46].

Nonetheless, biosensors based upon phage have the capability to find patterns in different major obstacles, distinguishing live cells of *Salmonella* quickly and efficiently, for example. In future, more novel and viable applications need to be developed for sensing *Salmonella* and other pathogens.

### 3.4. DNA-Based Biosensors for Sensing Salmonella

Genosensors are DNA biosensors that detect the analyte using hybridization reactions by detecting certain nucleic acids in bacterial cells [47]. They rely on the affinity and specificity of ssDNA for their own strand. The probes of nucleic acid are essential for recognizing the target in these biosensors. DNA recovered from the cells of *Salmonella* are usually denatured before being subjected to DNA probes. The sensor surface then undergoes hybridization, resulting in a quantifiable signal [48]. Das and his colleges (2014) used probes modified with ssDNA on screen-printed electrode (SPEs) for targeting the Vi genes of *Salmonella typhi* with LOD of 50 pM [49]. Various amplification techniques of DNA, including rolling circle amplification (RCA), are routinely combined into genosensors in order to achieve better sensitivity [50]. Nucleic acid probes are simple to design and modify, as well as flexible and thermally stable. However, they are mainly limited to genosensors. The key drawbacks of genosensors include their time-consuming genomic DNA extraction and fragmentation, requirement of skilled workers and need for signal amplifications.

Research on the detection of pathogens, mainly Salmonella, has progressed quickly in the last few years, emphasizing point-of-care applications. Antibodies and aptamers are undoubtedly among the best bio-receptors for the detection of *Salmonella* in on-field applications, but there has been surge in other bioreceptors, including peptides and genosensors.

## 4. Evaluation of Nanomaterials for Electrochemical Sensing

Nanomaterials provide a high specific surface area, allowing for a greater number of bioreceptors for immobilization. However, the immobilization of the conjugated antibodies and nanomaterials has been one of the major problems. The functionalization of nanomaterials is usually performed by non-covalent interaction including π–π stacking, electrostatic interaction or Van der Waals forces [51].

- Quantum-dots-based electrochemical biosensors

**Figure 3:**
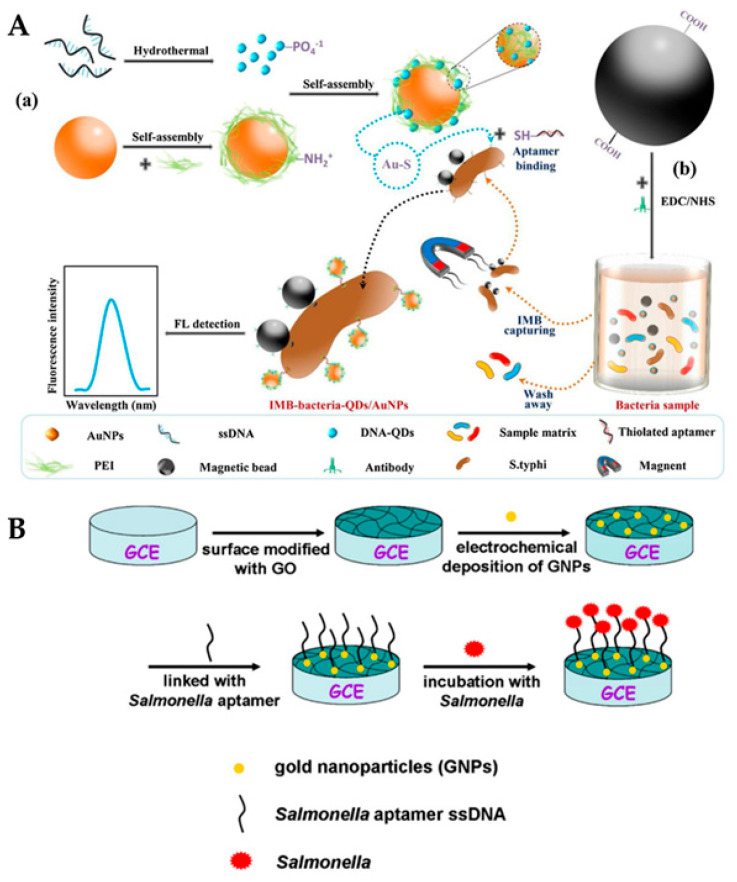
Nanomaterials (quantum dots and gold nanoparticles) for sensing. (**A**): (**a**) AuNPs and DNA-QDs for a self-assembled core–shell fluorescent probe. (**b**) *Salmonella typhimurium* was captured and enriched followed by isolation using magnetite microspheres. The attachment of surface-bound antibodies to the target bacteria and its reaction with the probe trailed by fluorescence measurements. Adapted with permission from Ref. [54]. Copyright 2020, Elsevier. (**B**): Detection of *Salmonella* using glassy carbon electrode (GCE). Graphene oxide was immobilized on the surface of GCE followed by electrodeposition of gold nanoparticles. Then, AuNPs were linked with ssDNA aptamers to capture the target *Salmonella* antigen. Adapted with permission from Ref. [70]. Copyright 2014, Elsevier.

Quantum dots have a broad absorption spectrum and a limited emission spectrum that varies in size. This phenomenon is caused by the semiconductor material’s variable band gaps for various nanocrystal sizes (the smaller the particle, the lower the band gap), which results in varied emission wavelengths from the electron–hole exciton recombination [52]. The availability of a wide variety of emission wavelengths from the QDs of various sizes allows for effective multiplexed analyses using traditional optical transduction [53]. Qi et al. combined DNA quantum dots with gold nanoparticles (AuNPs) (DNA-QDs) to form a fluorescent probe (DNA-QDs/AuNPs) that can be used with immunomagnetic separation (IMS) of *Salmonella typhimurium*. The probe polyethyleneimine (PEI)-coated AuNPs simplified the time-consuming process of aptamer modification and enhanced the fluorescence signal using electrostatic adsorption of DNA-QDs. *Salmonella typhimurium* was detected in a wide range of concentrations from 10 CFU/mL to 1.0 × 10^7^ CFU/mL, with a limit detection (LOD) of 13.6 CFU/mL, as shown in Figure 3A [54]. Non-radiative relaxation can occur if structural imperfections in the crystal lattice capture the departed electrons or holes [55]. To address this problem, core/shell composites containing a semiconductor material with a larger band gap range (often ZnS) were developed to passivate surface imperfections and photo-stability and improve quantum yields [56]. Core/shell QDs have become a potential alternative to organic fluorophores due to their great photochemical stability [57].

b. Magnetic nanoparticles (MNPs)-based electrochemical biosensors

Diagnostics, gene delivery, drug delivery, magnetic separation, in vivo imaging, and hyperthermia treatment are all made possible using MNPs. Furthermore, their ability to integrate into sensors makes them a perfect component of cutting-edge pharmacological and biological applications. Because of their durability and high sensitivity, MNPs-based electrochemical sensors have gained a lot of interest in biological, and pharmacological applications [58]. Magnetic nanoparticles offer an alternate to fluorescent labelling. Due to the reduced number of magnetic domains (regions of parallel oriented magnetic moments created by interacting unpaired electrons of an atom), nanosized magnetic nanoparticles exhibited distinct magnetic behavior compared to bulk material, resulting in superparamagnetic behavior. As long as no external magnetic field is supplied, such superparamagnetic behavior eliminates attraction or repulsive interaction between magnetic nanoparticles. The ability to concentrate the analyte before the detection event is a significant benefit of utilizing magnetic nanoparticles [59]. Magnetic nanoparticles customized with receptor units may be easily combined with the analyte. The nanoparticles agglomerate and separate after being exposed to an external magnetic field [60]. Devendra et al. investigated the Flagellin and MNP interactions with the antibody-immobilized sensor surface. A sequential two-step sandwich test and a preincubation one-step sandwich assay produced surface plasmon resonance (SPR) signal that was 7.5 times and 14.0 times greater than the direct assay, respectively. The assay’s detection limits in romaine lettuce samples and buffer were 5.2 log CFU/g and 4.7 log CFU/mL, respectively [61].

c. Carbon nanoparticles based electrochemical biosensors

Carbon nanotubes have an exceptional blend of nanowire shape, biocompatibility, and electrical characteristics. As a result, carbon nanotube interfaces have much improved capabilities, such as approaching the active areas of redox enzymes and wiring them to the bulk electrode [62]. Furthermore, its simple and well-documented organic functionalization provides nanostructured electrodes with novel features, such as particular biomolecule docking sites or redox mediation of bio-electrochemical interactions [63]. Furthermore, due to the spontaneous creation of extremely porous three-dimensional networks suited for the anchoring of a large number of bioreceptor units, CNT films have high electroactive surface areas, resulting in high sensitivities [64]. In a study by Subramanian et al., a disposable electrochemical immunosensor was developed for the simultaneous measurement of common foodborne pathogenic bacteria (*Salmonella*). The sensor was fabricated by attachment of anti-*Salmonella* antibodies on the surface of a multiwalled carbon nanotube-polyallylamine-modified screen-printed electrode. The suspension of bacteria bound to the immobilized antibodies when the sensor was incubated in liquid samples [65].

d. Metallic nanoparticles-based electrochemical biosensors

Characteristics including biocompatibility, electrical, and optical characteristics, ease of manufacture, and modification make gold nanoparticles the most often utilized noble metal nanoparticles for biosensor applications [66]. The optical behavior of gold surfaces, where irradiation with light of a given wavelength induces resonant surface plasmons, or oscillations of the electrons in the conduction band, is particularly noteworthy [67]. The oscillating electrons cannot travel down the surface when the particle size is substantially smaller than the incoming wavelength, as they do in traditional surface plasmon resonance (SPR) set-ups. Metallic nanoparticles are frequently used to anchor biorecognition components such as enzymes, antibodies, single-stranded DNA (ssDNA), RNA (ssRNA), and affibodies in biosensors [68]. The physicochemical features of MNPs have a big role in the efficacy and durability of these metabolic interactions [69]. Xiaoyuan et al. modified gold nanoparticles (AuNPs) and graphene oxide (GO) on the glassy carbon electrode (GCE) to enhance the electron transfer properties for the detection of *Salmonella typhimurium.* Upon addition of *Salmonella* to the reaction system, the current between the electrolyte and electrode reduced, leading to decrease in impedance and a detection limit of 3 CFU/mL being obtained, as shown in Figure 3B [70].

Integration of nanomaterials in biosensing platforms has boosted the limit of detection (LOD) tremendously in comparison with sensors especially with molecular probes. Additionally, nanomaterials act as surface modifiers, enhancing the conductivity and surface area, increasing the stability of the biosensor. The implementation of ultra-sensitive, stable nanomaterials in future would help in point-of-care applications. It would also help in detection of pathogens even before the symptoms appear, leading to an early mitigation of the disease.

## 5. Electrochemical Biosensors for *Salmonella* Detection

Sensors for the detection of *Salmonella* using the different bioreceptors, including various signal transducers processes, have increased their usage in recent years. Most of the sensors have been verified in food constituents, while others have the potential to be used in food samples. Different biosensing apparatuses for the detection of *Salmonella* have been addressed in this section, with a particular focus on electrochemical sensors. Their high sensitivity, low cost, and miniaturization potential make electrochemical biosensors the most commonly used sensing platform for the detection of *Salmonella*. Biosensors can be classified as amperometric, potentiometric, or conductometric based on the transducers used. A list of biosensors (electrochemical) for *Salmonella* detection strains are mentioned below in Table 1.

### 5.1. Amperometric Biosensors for Salmonella Detection

**Figure 4:**
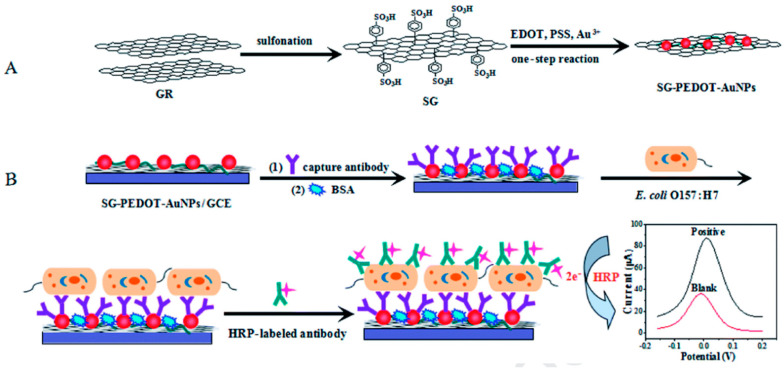
Principle of the sandwich assay. (**A**) Synthesis of sulfonated graphene (SG-PEDOT-AuNPs) composites and Poly-(3,4- 444 ethylenedioxythiophene) (PEDOT) in a single step reaction; (**B**) Schematic representation of electrochemical reaction where antibody was bound on the surface of glassy carbon electrode and detection of target by change in current. Adapted with permission from Ref. [82]. Copyright 2020, Elsevier.

Amperometry monitors variations in current when a constant voltage is applied for defined time. These sensors have acted as efficient models in the fast biosensing of *Salmonella* strains in food samples for decades, as shown in Figure 4 [82].

**Figure 5:**
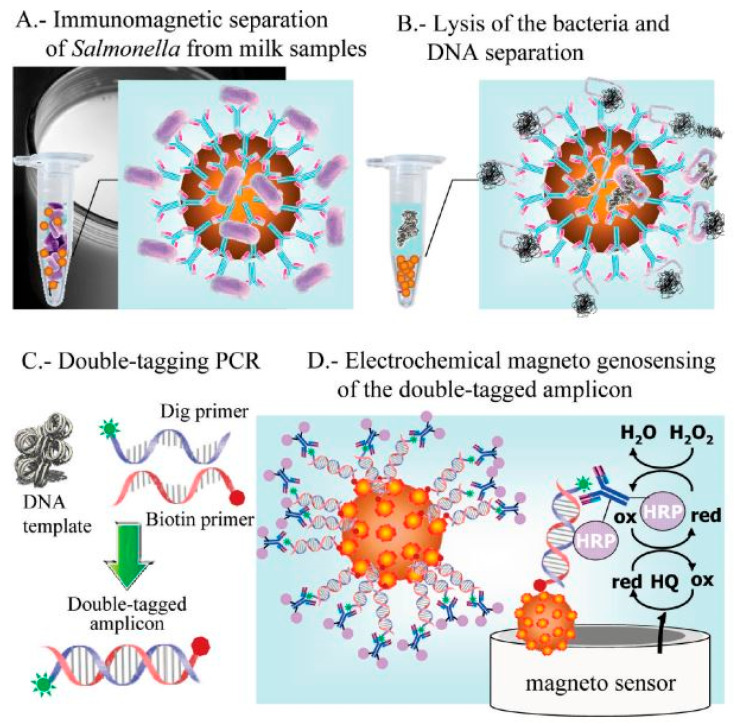
Electrochemical genosensing using immunomagnetic separation (IMS)/double-tagging PCR/m-GEC technique. (**A**) Separation of *Salmonella* contamination using Immunochromatographic separation in milk samples; (**B**) Bacterial lysis and separation of the nuclei acid; (**C**) Double-tagging of the nucleic acids; (**D**) Electrochemical genosensing (GEC) of double-tagged amplicon or the nucleic acid. Adapted with permission from Ref. [84]. Copyright 2009, American Chemical Society.

Tyrosinase carbon paste electrode was used to measure phenol concentrations in a chicken carcass using an indirect method for detecting *Salmonella typhimurium* in 2.5 h with a LOD of 5 × 10^3^ CFU/mL. The cells of *Salmonella typhimurium* were placed in between immunomagnetic beads (IMBs), and antibodies tagged with alkaline phosphatase (ALP). ALP was used as a catalyst for converting phenyl phosphate substrate to phenol, and tyrosinase carbon paste electrode used for the measurement. Furthermore, tyrosinase and horseradish peroxidase (HRP) electrode, which acted as a bio-enzyme, was used to enhance the LOD to 4.2 × 10^2^ CFU/mL 5 [83]. In a study by Susana et al., Salmonella typhimurium could be detected in food samples using magneto immunoseparation with an LOD of 1 CFU/mL as shown in Figure 5 [84]. 

In contrast to such indirect detection principles, the following research on *Salmonella* detection centered on a direct sandwich ELISA approach. To precisely capture *S. typhimurium*, Tothill and Salam (2009) used the surface of a screen-printed gold (SPG) electrode to adhere the antibodies. Following the addition of HRP-labeled antibodies, a sandwich structure was created. Using the enzyme mediator/substrate system as 3,3′,5,5′-tetramethylbenzidine (TMB)/H_2_O_2_, this method was able to diagnose *S. typhimurium* cells at a concentration of around 21 CFU/mL [85].

Later, Melo and colleagues (2018) doptimized several steps, including gold electrode pretreatment, antibody immobilization, enzyme–substrate, and concentrations of mediator with detection limit of 10 CFU/mL [86]. The amperometric method has been sensitive enough to detect *Salmonella*. Amperometric biosensors, on the other hand, rely on time-consuming labelling to boost the electrochemical response on the surface of electrode, limiting its use in the field.

### 5.2. Potentiometric Biosensors for Salmonella Detection

**Figure 6:**
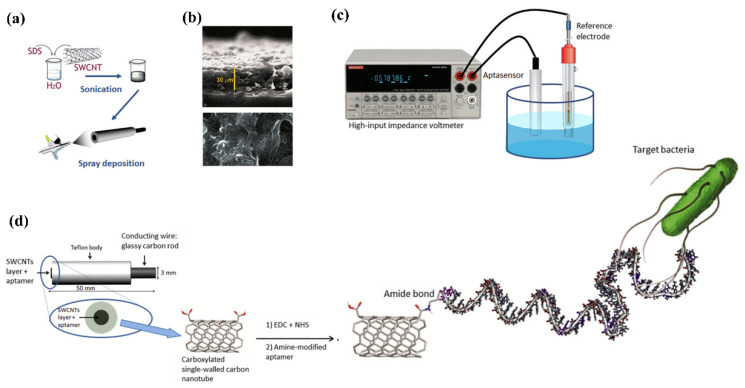
Fabrication of aptasensor on glassy carbon electrode (GCE). (**a**) Carbon nanotube deposition of on the surface of GCE; (**b**) Deposition of single-walled carbon nanotubes (SWCNTs) on GCE observed by (ESEM) environmental scanning electron microscope (representing a view from surface and lateral side). The surface view explains about the layer completely covering whole surface and the lateral view displays homogeneous height of the layer; (**c**) Potentiometric analysis; (**d**) Representation of an aptasensor for potentiometric analysis with specific aptamers functionalized with carboxylated SWCNTs and the correlation between the target bacteria and aptamer (Gustavo et al., 2013). Adapted with permission from Ref. [88]. Copyright 2013, Elsevier.

Under zero or insignificant current flow conditions, a high impedance voltmeter is used in potentiometric sensors for measurement of the change in electrical potential/electromotive force among the reference and working electrodes [87]. The cadmium ions amount produced during nanoparticle dissolution was used to measure the concentration of *S. typhimurium with* LOD of 20 CFU/mL in 75 min. The use of nanomaterials to generate/amplify the signal lengthens the time of analysis, and increases the assay’s complexity as shown in Figure 6 [88].

A label-free potentiometric detection was achieved in the presence of *S. typhi* with 0.2 CFU/mL in about 60 s. An important sensing technique of potentiometric sensors is based upon the surface blocking principle that has recently been presented for *Salmonella* detection, claiming to reach amplification capacities comparable to label-based techniques. The ion-flux internal marker was blocked via the detecting cell membrane attachment of *Salmonella*, resulting in a potentiometric response. *Salmonella* detection using these approaches helped in sensitive diagnosis with several CFU/mL of LODs within 1 h using transducers made up of paper-based strip electrode with a polymer inclusion membrane on an ion-selective electrode [89]. Exceptionally high sensitivity was obtained with the potentiometric biosensor. However, they have received less attention for *Salmonella* detection owing to the time-consuming tuning of experimental conditions and stabilization of the reference electrode.

### 5.3. Conductometric Biosensors for Salmonella Detection

Conductometry is a technique in which either no electrochemical reaction occurs at all on the electrodes or secondary reactions can be ignored. As a result, the conductivity of the electrolytic solution in the boundary layer is the most essential attribute in the conductometric approach, which varies according to a wide range of biological responses [90]. In comparison to other types of transducers, conductometric biosensors have several advantages such as the absence of the need for a reference electrode; operation at alternating voltage having a low amplitude, which helps prevent Faraday processes on electrodes; insensitivity to light, easy miniaturization, and integration using a low-cost thin-film standard technology [91]. First of all, they can be manufactured using low-cost thin-film standard technology. This, combined with the use of an improved approach for immobilizing biological material, leads to a significant reduction in both the primary cost of devices and the total cost of analyses. It is simple to use a differential measurement mode with inbuilt micro-biosensors, which compensates for external impacts and improves measurement accuracy significantly. The findings show that conductometric biosensors have a lot of promise [92]. Zarini et al. developed an electrochemical sandwich immunoassay and a reader was designed for signal measurement. The movement of bacteria was performed through electrical conductivity of the sensor with a detection limit of approximately 7.9 × 10^1^ CFU/mL within a 10 min process [93]. As this is still a relatively new trend in the field of biosensors, the development of commercial devices for point-of-care applications has a bright future.

## 6. New-Generation Sensing Platforms for *Salmonella*

Biosensors permitted sensitive and rapid detection of *Salmonella* in food; however, its on-site detection still required improvisation. The high cost, need for consistency, and the reproducibility of instrumentation are some of the major challenges. In the last few years, there has been a surge in the designing of multipurpose devices. Nanomaterials and advanced transdisciplinary research technologies have provided a new and significant direction for industrial applications. There has been an increase in the creation of on-field-suitable, customer friendly, software-based sensors for the sensitive and rapid detection of *Salmonella*.

### 6.1. Microfluidics Based Biosensing Platforms

**Figure 7:**
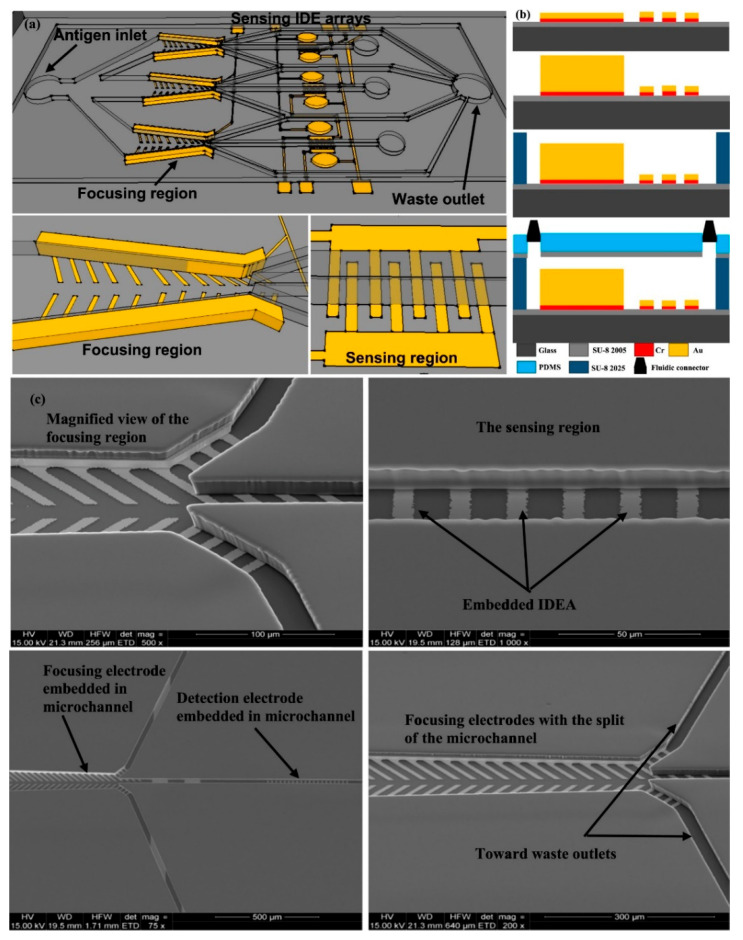
Impedance-based biosensor for Salmonella detection. (**a**) Representation of sensor based upon impedance, zoomed image of focusing and detection electrodes; (**b**) Image showing cross-sectional analysis of the layers fabricated in the biosensor including electroplated vertical walls, thin film electrodes with patterned structure, PDMS cover, fluidic connectors and SU8 2025 microchannel (**c**) SEM image of the fabricated device explaining the enlarged view of the detection region and focused region depicting the microchannel split. Adapted with permission from Ref. [100]. Copyright 2017, Elsevier.

The integration of various laboratory tasks into a small system is extremely desirable for on-site utilization of biosensing platforms. Microfluidics-based sensors that combine micro-channels for fluid conveyance with essential immunoassay components are gaining popularity [94,95,96]. These devices have an ability to regulate the flow of fluids in micro-channels using pressure and electro-kinetics, as well as execute a full analysis in a single chip, including sampling, separation, mixing, and detection [97]. It has several unique properties that benefit from the advantages of microfluidics, such as automation and downsizing capabilities, more efficient reagent usage, decreased time for processing, high-throughput analysis, and great mobility [98]. Electrochemical and optical detection are frequently combined with microfluidic devices for the creation of microfluidic-based sensing apparatuses for the detection of *Salmonella* [99]. Jasim et al. (2019) showed a microfluidic-based impedance biosensor that can detect multiple *Salmonella* serogroups in poultry products quickly and simultaneously. Three microchannels depicted in the gadget, each with a region to focus for sample concentrator, and a sensor space to detect bacterial cells. The main antigen inlet was used to inject poultry samples into the biosensor. The bacteria were then focused on the microchannel centerline and driven towards the sensor zone using positive dielectrophoretic force. Finally, an impedance analyzer was utilized for the detection of *Salmonella* in 40 min with a sensitivity of 7 CFU/mL, as shown in Figure 7 [100].

### 6.2. Internet of Things (IOT)-Supported Sensing of Salmonella

An IoT system is made up of sensors, and devices that communicate with the cloud via device-to-cloud communication model. Once the data reach the cloud, software analyze them and send an alarm or automatically alter the devices/sensors without any intervention from the user [101,102,103]. Food sensors incorporated in intelligent packaging provide retailers and customers with a quality indication [104]. The use of labels, such as a time-temperature indicator (TTI) that reflect a product’s accumulated time-temperature history, is a rudimentary kind of smart packaging. Different chemical substances, glucose, ethanol, or gas molecules, which commonly change the color to indicate the response, are utilized to test food quality using more advanced indicator sensors. A commercial tag or a chemical barcode, for example, may detect volatile amines in fish [105]. Sensors that measure contamination, bacterial content, color or texture deterioration, bruising, and other functions are available. Simple sensors, such as FoodScan, C2Sense, and the *Salmonella* Sensing System, are currently being prepared.

### 6.3. Clustered Regularly Interspaced Short Palindromic Repeats (CRISPR)-Based Electrochemical Sensors

**Figure 8:**
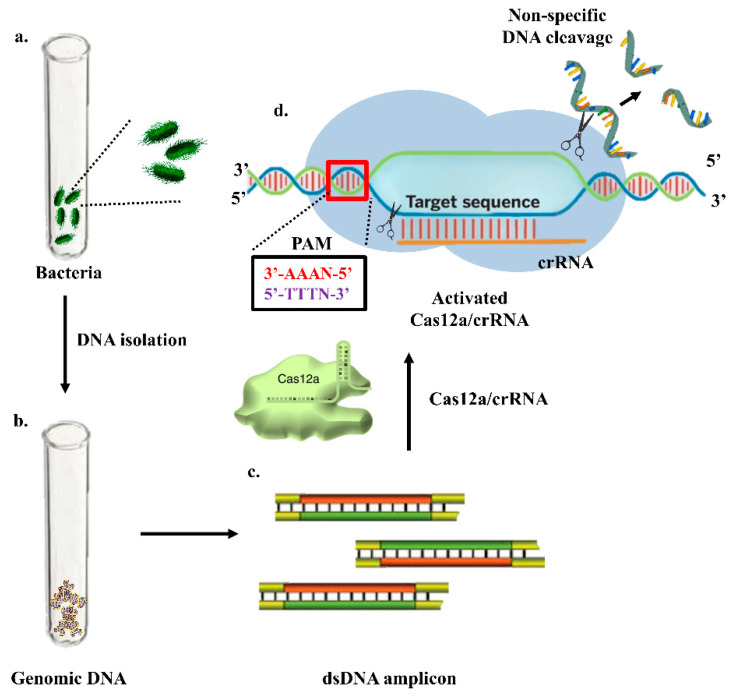
CRISPR based detection of *Salmonella*. (**a**) Bacterial culture; (**b**) Bacterial genomic DNA isolation; (**c**) dsDNA amplicon generation; (**d**) Binding of Cas12-crRNA complex with target DNA leading to trans-cleavage activity.

CRISPR is a genome editing tool used for the treatment of various diseases. However, with advancement in research, CRISPR has been utilized for the detection of pathogens. A specific gene of interest is detected using a specific guide and fluorescent or bioluminescent probe, as shown in Figure 8. Methods for the detection of *Salmonella* using CRISPR/Cas-based approaches have been developed recently, but these techniques are in the very early stage and require time for further advances. Cas9, Cas12 and Cas 13 proteins are mainly used in the detection of pathogens. The CRISPR/Cas9 system is based on the nucleolytic activity of the endonuclease protein, Cas9, which is guided to the desired site in the genome by a specificity determinant RNA, known as the guide RNA (gRNA). In addition, another sequence—known as the protospacer-adjacent motif (PAM), present adjacent to the target site—is recognized by the CRISPR/Cas9 system and is crucial for the functionality of Cas9. The Cas9 protein binds to the target location in the presence of gRNA with high precision and performs a double strand break followed by the introduction of indel mutations at the cleavage site by using the non-homologous end joining repair mechanism of the cell [106]. Cas12 targets DNA and trans-cleaves single-stranded DNA probes [107]; Cas13 targets the RNA sequence guided by a crRNA. Additionally, Cas13 boasts unique collateral cleavage activity; collateral cleavage of a fluorescent reporter detects the presence of the target sequence in sample RNA [108]. For pathogen identification, several methods have been used, the majority of which are PCR-based; nevertheless, these require expensive chemicals and equipment, as well as high levels of working expertise. Clustered regularly interspaced short palindromic repeats (CRISPR)/CRISPR-associated protein (Cas) systems have been employed in genome editing because of their capacity to accurately detect and cleave particular genome sequences [109]. Furthermore, after recognizing the target, CRISPR/Cas systems, including as Cas12a, Cas13, and Cas14 orthologues, display non-specific enzymatic activities that can be used for the detection of nucleic acid, such as degradation of a tagged nucleic acid to create a fluorescence signal. Multiplexing is feasible with CRISPR/Cas systems, leading to a diagnostic test for the identification of more than one target [110]. Developing devices that combine lateral flow systems with CRISPR/Cas might lead to low-cost, accurate, and extremely sensitive diagnostics that can be deployed in the field. From agriculture to human health, these sensors have a wide range of uses. Electrochemical CRISPR (E-CRISPR)-integrated point-of-care sensors (POCs) are the need of the hour due to their diverse benefits and ease of translation into smart devices, allowing physicians to analyze the data in less time and provide better patient care. [111,112]. Song et al. transcribed the target DNA into RNA T7 transcriptase and stimulated the RNase activity of the Cas13a protein. The active Cas13a protein cleaved the self-folding quenched fluorescence probe to generate the fluorescent signal [113]. The integration of CRISPR/Cas technology in electrochemical sensors would help in the detection of pathogens at a genomic level. It would facilitate the detection of pathogens at very low concentrations, leading to early treatment. The technology for the detection of *Salmonella* using CRISPR is at a very early stage of development, and requires a proper laboratory set up with the required equipment for commercialization.

## 7. Challenges in Development of Electrochemical Sensors against *Salmonella*

Biosensors have gained a lot of attention for *Salmonella* detection as they offer huge potential in the field of food analysis. However, they are not yet ready to be utilized as a regular analytical tool in food corporations because the technology is in its infancy. The major obstacles faced in the development of electrochemical sensors are as follows: (1) achieving a low limit of detection (LOD); (2) nullifying non-specific adsorption of interfering species; (3) on-site detection of samples, or conversion of a sensor into a point-of-care device; (4) preserving the sensor’s stability and repeatability in complicated actual matrices [114]. Furthermore, when the analyte is a biological sample, i.e., bacteria, differentiation between living and dead cells is a major challenge. Some of the obstacles that need to be overcome in future are mentioned below.

The detection of Hg^2+^ was possible up-to picomolar level using silver nanoparticles modified with a glassy carbon electrode [115]. Nonetheless, these altered surfaces provide a challenge, since they are not always as repeatable as assumed. Because controlling the synthesis and immobilization of nanoparticles with varied populations of size and shape is challenging, conformation and the topology of these nanomaterials may change across each sensor. Under different environmental circumstances, nanoparticles have a tendency to change their behavior. The repeatability of the sensor that develops as a result of the increasing complexity of the different surfaces are a trade-off. Because it is impossible to evaluate every sensor generated in mass-production facilities, sensor-to-sensor repeatability is critical during the manufacturing process. Instead, when the sensors are manufactured, statistical sampling of a portion of the sensors is carried out, and the testing and calibration findings should be relevant to the entire batch [114]. Hydrogels, rubber-like composites, new polymers and organo-gels are often used to create tiny sensors with high stability. When these very stretchy materials are combined with nanomaterials that have exceptional electrical conductivity, the sensors demonstrate great analytical performance [116,117].

Electrochemical sensors have shown considerable promise as diagnostic tools. The issues stated above, however, are applicable not just to clinical applications, but also to other industries such as food safety, environmental monitoring, forensic investigation, defense, military applications, agriculture, and the electronics industry. Fortunately, the breakthrough, made possible by the use of nanomaterials, is a huge step forward that affects all of the sectors described above [114].

## 8. Conclusions

Although of the applications of biosensing techniques for the detection of *Salmonella* in the food industry are in their early stages of development, especially when compared to conventional techniques, they are promising forms of technology for the creation of biosensing platforms for on-site use. Biosensing methods, in combination with nanomaterials and innovative bioreceptors, will continue to be appealing in the future with many intriguing possibilities. To manufacture biosensors with more integrated, practical, portable qualities, and automated use, increased attention must be paid for the implementation of new biosensing approaches with automated and portable instruments, including smartphones and microfluidic devices. Furthermore, as information technology advances, sensors combined with big data analytics and artificial intelligence (AI) are anticipated to revolutionize the technology, aiding effective monitoring strategies and predicting contaminations in the food sector.

## Figures and Tables

**Table 1:** Electrochemical biosensors for the detection of *Salmonella* strains.

Sl No.	Serotype	Bio-Recognition Element	Detection Method	Range of Detection	LOD	References
1.	*Salmonella typhimurium*	Aptamer	Potentiometry	67–6.7 × 10^5^ CFU/mL	10 CFU/mL	[71]
2.	*Salmonella typhimurium*	Aptamer	EIS	10–10^8^ CFU/mL	6 CFU/mL	[72]
3.	*Salmonella typhimurium*	Antibody	DPV	10–10^7^ CFU/mL	3 CFU/mL	[73]
4.	*S. pullorum & S. gallinarum*	Antibody	CV	10^1^–10^9^ CFU/mL	1.61 × 10^1^ CFU/mL	[74]
5.	*S. enteritidis*	Genosensor	SWAS V	50 pg/mL–50 ng/mL	0.5 ng/mL	[75]
6.	*S. pullorum*	Antibody	EIS	10^2^ to 10^6^ CFU /mL	89 CFU /mL	[76]
7.	*S. pullorum and S. gallinarum*	Antibody	CV	10^4^–10^9^ CFU/mL	3.0 × 10^3^ CFU/ mL	[77]
8.	*Salmonella typhimurium*	Antibody	EIS	10–10^6^ CFU/mL	10 CFU/mL	[78]
9.	*Salmonella typhimurium*	Aptamer	DPV	20–20^7^ CFU/mL	16 CFU/mL	[79]
10.	*Salmonella enterica*	Aptamer	DPV	10–10^6^ CFU/mL	10 CFU/mL	[80]
11.	*Salmonella typhimurium*	Antibody	DPV	10–10^5^ CFU/ml	5 CFU/mL	[81]

DPV: Differential Pulse Voltammetry, CV: Cyclic Voltammetry, EIS: Electrochemical Impedance Spectroscopy, SWAS V: Square wave Voltammetry.

## Data Availability

Not Applicable.
